# Electronic public health surveillance in developing settings: meeting summary

**DOI:** 10.1186/1753-6561-2-s3-s1

**Published:** 2008-11-14

**Authors:** Jean-Paul Chretien, Sheri H Lewis

**Affiliations:** 1 Department of Defense Global Emerging Infections Surveillance and Response System (DoD-GEIS), Silver Spring, Maryland, USA; 2 Johns Hopkins University Applied Physics Laboratory, Laurel, Maryland, USA

## Abstract

In some high-income countries, public health surveillance includes systems that use computer and information technology to monitor health data in near-real time, facilitating timely outbreak detection and situational awareness. In September 2007, a meeting convened in Bangkok, Thailand to consider the adaptation of near-real time surveillance methods to developing settings. Thirty-five participants represented Ministries of Health, universities, and militaries in 13 countries, and the World Health Organization (WHO). The keynote presentation by a WHO official underscored the importance of improved national capacity for epidemic surveillance and response under the new International Health Regulations, which entered into force in June 2007. Other speakers presented innovative electronic surveillance systems for outbreak detection and disease reporting in developing countries, and methodologies employed in near-real time surveillance systems in the United States. During facilitated small- and large-group discussion, participants identified key considerations in four areas for adapting near-real time surveillance to developing settings: software, professional networking, training, and data acquisition and processing. This meeting was a first step in extending the benefits of near-real time surveillance to developing settings. Subsequent steps should include identifying funding and partnerships to pilot-test near-real time surveillance methods in developing areas.

## Background to the meeting

Public health surveillance is the systematic, ongoing assessment of the health of a community, based on the collection, interpretation, and use of health data and information [1]. Public health agencies conduct surveillance to identify and characterize health-related problems and evaluate interventions. In recent years, technological and analytical innovations facilitated public health surveillance systems designed for rapid identification of communicable disease epidemics. These systems monitor pre-diagnostic information – such as patient syndromes (i.e., constellations of signs and symptoms, such as "influenza-like illness" defined by fever and specific respiratory symptoms) or chief complaints – to detect outbreaks before traditional surveillance approaches based on more definitive diagnoses, and to provide near-real time situational awareness during outbreaks or heightened concern for outbreaks [2,3].

Most of these systems are currently deployed in high-income countries. This is understandable, as the hardware, software, communication systems, and human resources needed to operate them are not widely available in developing countries. If the tools and approaches of near-real time surveillance could be adapted to low-resource settings, though, they could help health authorities address surveillance challenges [4]. For example, syndromic surveillance could detect emerging disease outbreaks before diagnostic criteria are established; automated data processing could reduce human workload; and objective criteria for assessing morbidity trends could help central offices aggregate and interpret surveillance results across healthcare facilities.

On September 11–12, 2007, a meeting convened in Bangkok, Thailand, to consider how the tools and approaches of near-real time surveillance could be adapted to developing settings [5]. The workshop was organized by the Johns Hopkins University/Applied Physics Laboratory (JHU/APL) and U.S. Department of Defense Global Emerging Infections Surveillance and Response System (DoD-GEIS), and hosted by the Armed Forces Research Institute of Medical Sciences (AFRIMS), a joint Royal Thai Army-U.S. Army center in Bangkok. There were 35 participants representing Ministries of Health, universities, and militaries in 13 countries (Table 1), and the World Health Organization (WHO).

**Table 1:** Meeting participants' countries

**Asia**
Cambodia
Hong Kong SAR
Indonesia
Malaysia
Nepal
Philippines
Thailand
Vietnam
**Middle East**
Israel
Lebanon

**South America**
Peru

**North America**
Canada
United States

## Overview of presentations

Guénaël Rodier (Director, International Health Regulations Coordination, WHO), in the keynote presentation, introduced the new International Health Regulations. IHR (2005), which entered into force in June 2007, requires WHO member states to strengthen their existing capacity for disease surveillance and response [6]. While the previous Regulations covered only four specific diseases (cholera, plague, yellow fever, and smallpox), IHR (2005), addressing the threat of new and emerging diseases, requires countries to notify WHO of any events that may constitute a public health emergency of international concern. The presentations and discussions that followed Dr. Rodier's presentation considered how near real-time surveillance could improve outbreak detection and public health situational awareness in developing countries.

Three speakers described national systems for notifiable disease reporting and outbreak detection. Soawapak Hinjoy and Rungnapa Prasanthong presented Thailand's communicable disease surveillance program, which tracks 70 conditions. At healthcare facilities, patient data are entered into computer using a standardized information system (at health centers) or various software (at hospitals), and emailed to district health offices. At district and higher-level offices, epidemiological software helps assess trends and identify outbreaks. The system facilitates timely outbreak detection by including syndromes and provisional diagnoses given before laboratory confirmation (laboratory results may be added to the database later). Challenges include under-utilization of data and internet access problems at sub-national levels.

Nada Ghosn described Lebanon's notifiable disease surveillance program, focusing on 4 diseases for which rapid detection is especially important [7]. Public and private hospitals report cases of hepatitis A, brucellosis, amebic dysentery, and typhoid fever monthly, by mail, through provincial offices to the Ministry of Health. There, data are entered into computer and epidemiological software is used to compare them to historical levels, generating alerts when thresholds are met. While alerts have high positive predictive value for verified outbreaks, they often occur weeks after outbreaks begin. The Ministry of Health is considering electronic data entry at hospitals to speed data transmission and public health response.

Fadzilah Kamaludin described two complementary systems of Malaysia's Ministry of Health. The notifiable disease reporting system, which had used telephone, fax, or mail to transmit data from hospitals through district offices to the Ministry of Health, instituted electronic data entry at district offices in 2004. This sped data transmission and analysis, but rapidly adding new case definitions remained difficult. The Ministry of Health thus initiated surveillance for six syndromes likely to capture patients with emerging, epidemic-prone diseases. A recent evaluation found the syndromic surveillance system's fax-based reporting cumbersome for hospital staff, and therefore recommended electronic data entry for this system as well.

Three speakers described systems designed specifically for rapid outbreak detection. Jariyanart Gaywee presented a system developed by AFRIMS for use by Royal Thai Army units in remote border areas near Cambodia, Myanmar, and Malaysia. Each day, units send information on diseases of military importance (such as malaria and respiratory illness) by radio or fax to their regional headquarters, where data are entered into computer and emailed to AFRIMS for analysis. The system has high acceptability in the Royal Thai Army. Challenges include internet access problems and frequent rotation of soldiers who operate the system, requiring frequent training of new personnel.

Hadi Siswoyo presented the Early Warning Outbreak Recognition System (EWORS) developed by the Indonesian Ministry of Health and U.S. Naval Medical Research Unit-2 (Jakarta) [8]. Selected provincial hospitals collect sign and symptom data from patients with suspected acute infection and enter the data into computer using EWORS software, which facilitates comparison to historical values for outbreak detection. Data also is emailed to the central hub for analysis daily. The Ministry of Health has found EWORS especially useful in detecting and tracking dengue and diarrhea outbreaks, but would prefer more frequent and thorough analysis at EWORS sites to complement central analysis.

Giselle Soto reported an evaluation of Alerta, a Peruvian Navy system designed by U.S. Naval Medical Research Center Detachment (Lima) and Voxiva [9]. Alerta allows data transmission by internet or mobile phone from surveillance sites to the system hub (reporting is weekly or daily, depending on the disease or syndrome). Alerta facilitated response to outbreaks that otherwise would not have been identified quickly, including at remote Amazon bases, where data transmission using cellular networks has proven especially useful. Challenges after 4 years of operation include turnover of trained personnel, conducting regular and efficient monitoring and evaluation, and incorporation of laboratory data.

The final three speakers provided overviews of methodologies common in syndromic surveillance systems. Sheri Lewis identified critical considerations in selecting data sources for surveillance systems [10]. Kieran Moore described how the software interface and visualization tools can improve data interpretation, drawing on examples from several U.S.-based syndromic surveillance systems [11]. Andres Lescano [12] presented the key analytic methods for syndromic surveillance systems, including statistical methods for both outbreak detection and system performance monitoring.

## Overview of discussion

Participants divided into several small groups for facilitated discussion of three broad questions: 1) What is the current state of outbreak surveillance in participants' countries? 2) How could electronic systems enhance outbreak surveillance? 3) What is needed to achieve this improved state, and what are the obstacles? Facilitators summarized each group's session for the full group of meeting participants, which then discussed the small group results.

Through the small- and large-group discussions, meeting participants identified key considerations in 4 areas for adapting near-real time surveillance methods to developing settings:

### Software

Several participants suggested a need for more open-source software that Ministries of Health could adapt to their particular surveillance needs. Participants using various commercial software for transmitting, visualizing, and analyzing surveillance data found it effective but identified recurring licensing costs and an incomplete sense of ownership (since the supplier provides enhancements or adaptations) as significant limitations. Participants identified several freely available software packages used in syndromic surveillance systems that might be adapted to developing settings, and felt that partnerships between system developers in resource-limited areas and experienced syndromic surveillance practitioners would facilitate the adaptation.

### Professional networks

There was general agreement that innovative surveillance practitioners in developing countries would benefit from stronger professional networking, so that they could learn from each other's experience and share ideas. A web-based forum was suggested as a way to facilitate international networking. It was noted that collecting surveillance "success stories" through such networking could help practitioners communicate the importance of their work to policy-makers and sponsors.

### Training

The need for frequent training because of personnel turnover and attrition was a common challenge. One suggestion for achieving better competence for public health practice, including surveillance, in the healthcare workforce was to incorporate basic training into medical and nursing school curricula, thereby reducing requirements for on-the-job training. Several participants suggested that Ministries of Health include private sector healthcare providers when conducting surveillance-response continuing education campaigns, not just public hospitals.

### Data collection and processing

Participants noted that many Ministries of Health require collection of data that could be used for effective syndromic surveillance, if reporting were timelier. For example, some syndromic surveillance systems in the United States and elsewhere define syndromes using International Classification of Diseases (ICD) codes, which are reported by hospitals in many countries. Electronic data entry during initial data collection was identified as a key facilitating factor in making data available for analysis in near-real time. Various platforms for collecting and transmitting patient data were discussed, including personal digital assistants, mobile phones, and laptop computers.

## Future directions

By bringing together public health practitioners from countries with limited resources for surveillance and countries with well-funded surveillance efforts, the meeting reported here could be a first step in extending the benefits of near-real time surveillance to developing settings. Subsequent steps should include identifying funding and partnerships to pilot-test surveillance approaches or tools, such as training methods, software, and data collection platforms, for improving outbreak detection and public health situational awareness in developing areas. Obstacles to implementing such innovations are significant, but as the experience of some meeting participants proves, not insurmountable.

## Competing interests

The authors declare that they have no competing interests.
